# Sigmoid colocolic fistula caused by intrauterine device migration: a case report

**DOI:** 10.1186/1752-1947-8-81

**Published:** 2014-03-04

**Authors:** Amila Weerasekera, Pravin Wijesinghe, Nilhan Nugaduwa

**Affiliations:** 1 Base Hospital Wathupitiwala, Nittambuwa, Sri Lanka

**Keywords:** Fistula, Intrauterine device, Laparoscopy, Laparotomy, Migration, Perforation

## Abstract

**Introduction:**

The intrauterine device is a form of contraception with a long duration of action and few systemic side effects. Migration into the abdominal cavity may occur early or years after insertion giving rise to bowel obstruction, perforation, ischemia, mesenteric injury, strictures or fistulae. Colocolic fistula formation is a rare but serious complication of intrauterine device migration, which may lead to difficulties in diagnosis and device retrieval.

**Case presentation:**

We report the case of a 29-year-old Sri Lankan woman who became pregnant 5 years after intrauterine device insertion. The device could not be located during pregnancy. She was asymptomatic and defaulted follow up during the antenatal period. She had an uncomplicated vaginal delivery. A subsequent laparotomy for device retrieval failed due to technical difficulties. A repeat laparotomy identified a sigmoid colocolic fistula with adhesions to the fallopian tube. The device was removed and colonic defects primarily closed following which the patient made an uneventful recovery.

**Conclusions:**

All translocated intrauterine devices should be removed regardless of type and location. This case illustrates that they may cause complex bowel lesions leading to serious technical difficulties during retrieval. With the increasing use of minimally invasive approaches for intrauterine device retrieval, a low threshold for open surgery in complicated cases is advocated.

## Introduction

The intrauterine device (IUD) is a form of contraception with a long duration of action and few systemic side effects, but it can cause significant morbidity following migration into adjacent organs. Involvement of small and large bowel is known to cause obstruction, perforation, ischemia, mesenteric injury, strictures and fistulae [1]. Pregnancy following IUD migration leads to difficulty in localization and removal of the device. We report the case of a Sri Lankan woman who became pregnant after IUD insertion and was found to have a sigmoid colocolic fistula caused by device migration.

## Case presentation

A 29-year-old Sri Lankan woman presented at a period of gestation of 12 weeks in her third pregnancy, 5 years after insertion of a copper T-380A IUD. During pregnancy, the locator strings could not be identified and the device was not visualized by transabdominal or transvaginal ultrasound. There were no clinical features suggestive of perforation or intra-abdominal bleeding. As she was asymptomatic, further investigations and device retrieval were deferred. She was then lost to follow up until 38 weeks, when she was admitted to a peripheral hospital and underwent a vaginal delivery with no complications.

**Figure 1:**
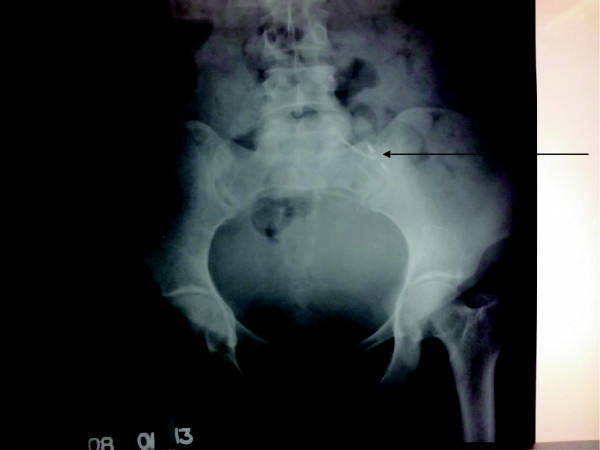
Anteroposterior X-ray of pelvis showing the intrauterine device (arrowed).

Following delivery, she was investigated to locate the missing IUD. X-rays showed the device in the pelvic cavity (Figure 1). As she had repeatedly defaulted follow up by health care services, it was unlikely that she would comply with plans for evaluation and device retrieval at a later date. Early intervention was therefore planned in the same unit. She underwent a laparotomy on day two postpartum, during which a mass involving her sigmoid colon was found in her pelvis. The IUD was palpable within the mass. The extent of bowel involvement was difficult to assess, and access was difficult due to the large puerperal uterus. Retrieval was therefore abandoned and she was transferred to a high-volume surgical unit.

**Figure 2:**
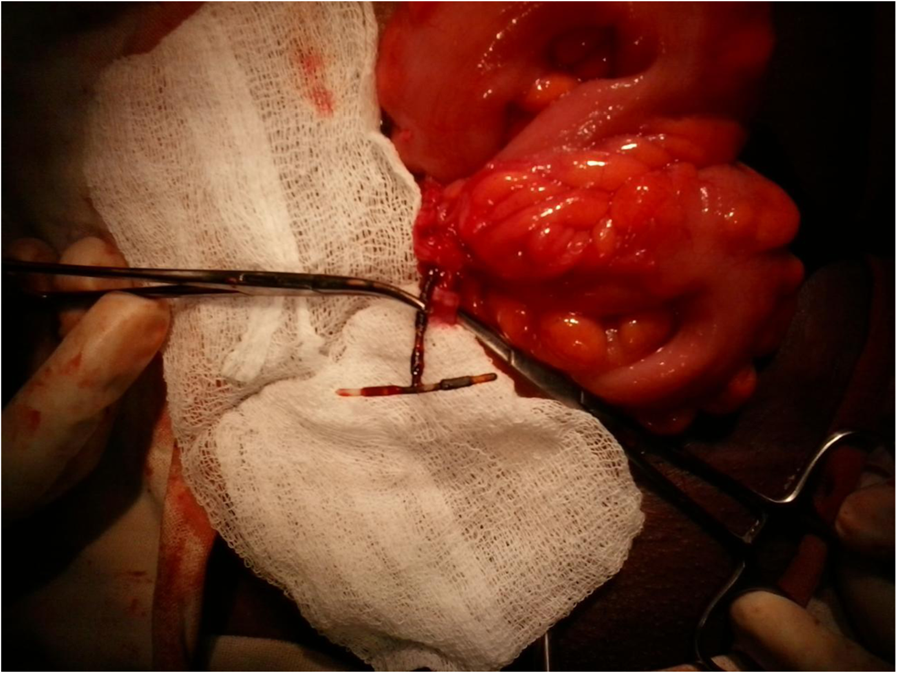
**The sigmoid colon was delivered through a transverse incision.** The intrauterine device was removed from the proximal and distal sigmoid. Part of the stem is seen within the fibrous tract extending from the uterus to the colon.

**Figure 3:**
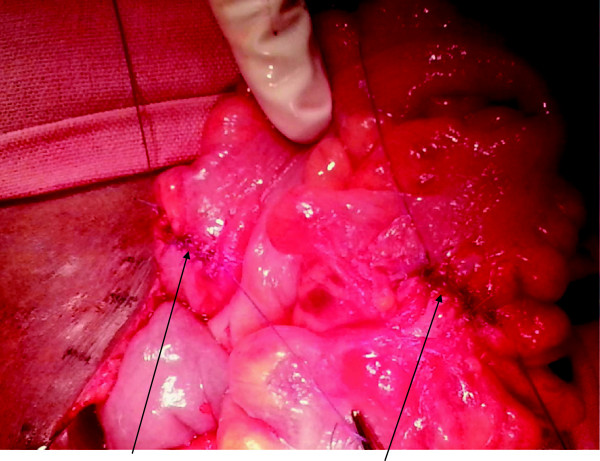
**Removal of the intrauterine device colon left two openings in the proximal and distal sigmoid colon.** The edges were trimmed and both defects primarily sutured (arrowed).

A repeat laparotomy was planned with mechanical bowel preparation and consent for a stoma, in anticipation of a difficult dissection. Under prophylactic antibiotic cover, her suprapubic transverse incision was reopened and her pelvis explored. The IUD was identified with the two arms forming a fistula between proximal and distal parts of her sigmoid colon. The protruding stem was embedded in a mass of fibrous tissue extending from her left fallopian tube to her sigmoid colon. The device was removed (Figure 2) and the fistula tract and fibrous tissue containing the stem were excised. The openings of the fistula in the proximal and distal sigmoid were about 1cm in diameter. These were primarily closed with serosubmucosal sutures (Figure 3). Postoperative recovery was uneventful.

## Discussion

The incidence of uterine perforation by IUDs is reported to be between 1.3 and 1.6 per 1000 insertions [1]. This may result in device migration into adjacent structures including the urinary bladder, bowel, omentum and retroperitoneum [2]. Factors affecting migration include uterine size, position, timing of the insertion, congenital uterine anomalies and previous surgery [3]. If inserted during the puerperium, uterine involution, strong contractions and soft consistency of the uterus may increase risk of perforation [3]. Presentation to healthcare may be early or late; a Swedish survey showed that a majority of early presentations (that is, within 1 month) were due to lower abdominal pain, whereas patients diagnosed later were mostly asymptomatic [4].

The commonest sites for intestinal perforation are the sigmoid colon, small intestine and rectum [5]. Both early and late bowel perforation have been reported [6]. Early puerperal insertion and subsequent pregnancy appear to be risk factors for bowel injury [7]. The device may be partially or completely embedded in the bowel wall [5]. A case of colocolic fistula formation by a Multiload Cu250 device has previously been reported by Pirwany and Boddy [8]. Bowel perforation may be asymptomatic or present with abdominal pain, peritonitis, subacute intestinal obstruction or as strings at the anus [2]. In asymptomatic patients, migrating IUDs may remain undetected for years [5].

As patients are not routinely followed up, migration may be suspected only due to a subsequent pregnancy. Device localization during pregnancy may be difficult due to radiation risk limiting use of X-rays and computed tomography. Visualization by ultrasound is poor due to surrounding bowel loops [2]. Magnetic resonance imaging may be performed if specific recommendations given by the manufacturer are followed [9]. In asymptomatic patients diagnosis and retrieval may be safely delayed until delivery. Problems in the puerperal patient include difficulty in surgical access caused by the large uterus. This may be avoided by delaying the procedure until the uterus has involuted.

World Health Organization guidelines recommend removal of migrating IUDs irrespective of their type and location [10]. Copper-containing devices, which are widely used, are known to cause considerable inflammation and a marked omental response [11]. The resulting fibrosis may make device retrieval technically difficult.

Colonoscopic [7] and laparoscopic [12] techniques are increasingly being used for retrieval of IUDs. Colonoscopy is useful in cases where the device is within the lumen or embedded in the inner part of the wall. Colonoscopic retrieval may lead to difficulties if the device is partly embedded in adjacent structures. A complex lesion such as a colocolic fistula would be extremely difficult to identify at colonoscopy. This approach could lead to intraperitoneal leaks from either lumen which would be difficult to detect through the scope. Undetected perforations and subsequent peritonitis would be life threatening.

Possible laparoscopic options for IUDs embedded in the bowel include device extraction and intracorporeal suturing, or resection of the affected segment with primary anastomosis [13]. The advantages of laparoscopic approaches include: reduced tissue trauma, lower postoperative pain, early return to function and lower risk of intra-abdominal adhesions.

However, laparoscopic retrieval has had variable outcomes, with reports of repeat laparoscopy [5], conversion to laparotomy [5,8,14] and colostomy [14]. In a systematic review by Gill *et al.* the major indications for conversion to laparotomy were adhesions and bowel perforation [5]. For all translocated intra-abdominal devices, the laparotomy rate following diagnostic laparoscopy was 34.6% in this study. However, for devices embedded in the bowel the rate was as high as 68%.

Other concerns about laparoscopy include transmission of thermal energy by the copper IUD [5], which may cause undetectable visceral damage during laparoscopy. Therefore a harmonic scalpel may be used for dissection of an IUD off visceral structures. In the presence of a complicated lesion such as a colocolic fistula, laparoscopic dissection and delineation of the anatomy would be technically demanding. A safe approach would be to locate the device using laparoscopy, with a low threshold for conversion to open surgery in difficult cases.

## Conclusions

Insertion of any IUD should be performed only by a specialist and according to the latest instructions. A translocated IUD may remain asymptomatic and undetected, but should be removed irrespective of the type and location due to risk of serious complications. A missing device during pregnancy poses challenges in localization and retrieval. This case highlights the fact that migrating IUDs may cause complex lesions involving the bowel leading to serious technical difficulties during device retrieval. With the increasing use of minimally invasive approaches for IUD retrieval, a low threshold for open surgery in complicated cases is advocated.

## Consent

Written informed consent was obtained from the patient for publication of this case report and accompanying images. A copy of the written consent is available for review by the Editor-in-Chief of this journal.

## Abbreviations

IUD: Intrauterine device.

## Competing interests

The authors declare that they have no competing interests.

## Authors’ contributions

AW gathered data and prepared the initial manuscript. AW performed the surgery and PW made critical revisions to the manuscript. All authors were involved in the surgical care of the patient. NN supervised the project overall. All authors read and approved the manuscript.
